# Response of Soil Microbiota, Enzymes, and Plants to the Fungicide Azoxystrobin

**DOI:** 10.3390/ijms25158104

**Published:** 2024-07-25

**Authors:** Małgorzata Baćmaga, Jadwiga Wyszkowska, Jan Kucharski

**Affiliations:** Department of Soil Science and Microbiology, University of Warmia and Mazury in Olsztyn, 10-727 Olsztyn, Poland; m.bacmaga@uwm.edu.pl (M.B.); jan.kucharski@uwm.edu.pl (J.K.)

**Keywords:** azoxystrobin, ecotoxicology, enzymes, microorganisms, plants, soil

## Abstract

The present study was aimed at assessing the impact of azoxystrobin—a fungicide commonly used in plant protection against pathogens (Amistar 250 SC)—on the soil microbiota and enzymes, as well as plant growth and development. The laboratory experiment was conducted in three analytical terms (30, 60, and 90 days) on sandy clay (pH—7.0). Azoxystrobin was applied to soil in doses of 0.00 (C), 0.110 (F) and 32.92 (P) mg kg^−1^ d.m. of soil. Its 0.110 mg kg^−1^ dose stimulated the proliferation of organotrophic bacteria and actinobacteria but inhibited that of fungi. It also contributed to an increase in the colony development index (CD) and a decrease in the ecophysiological diversity index (EP) of all analyzed groups of microorganisms. Azoxystrobin applied at 32.92 mg kg^−1^ reduced the number and EP of microorganisms and increased their CD. PP952051.1 *Bacillus mycoides* strain (P), PP952052.1 *Prestia megaterium* strain (P) bacteria, as well as PP952052.1 *Kreatinophyton terreum* isolate (P) fungi were identified in the soil contaminated with azoxystrobin, all of which may exhibit resistance to its effects. The azoxystrobin dose of 0.110 mg kg^−1^ stimulated the activity of all enzymes, whereas its 32.92 mg kg^−1^ dose inhibited activities of dehydrogenases, alkaline phosphatase, acid phosphatase, and urease and stimulated the activity of catalase. The analyzed fungicide added to the soil at both 0.110 and 32.92 mg kg^−1^ doses inhibited seed germination and elongation of shoots of *Lepidium sativum* L., *Sinapsis alba* L., and *Sorgum saccharatum* L.

## 1. Introduction

Pesticides underpin the maintenance of plant quality and health through their key role in eradicating diseases, pests, and weeds [1,2,3,4]. Azoxystrobin is a fungicide from the group of strobilurins, extensively used in agricultural production due to its broad spectrum of effects and high efficacy against fungal pathogens of crops [5,6,7]. Strobilurin was originally isolated from the fungus *Strobilurus tenacellus* in 1977, whereas azoxystrobin was introduced on the German market in 1996 [8]. Edwards et al. [9] have pointed out that the half-life of azoxystrobin in soil spans from 14 days to 6 months, which is related to the activity of soil microorganisms and enzymes. In addition, azoxystrobin is a fungicide from the group of external quinone inhibitors that inhibit mitochondrial respiration by blocking the transfer of electrons in the cytochrome bc1 complex. Moreover, it inhibits the oxidation of nicotinamide adenine dinucleotide (NADH) and adenosine triphosphate (ATP) [7,8,10,11] and exerts multifaceted effects on *Ascomycetes*, *Basidiomycetes*, and *Oomycetes* fungi. Its EC_50_ (half-maximal effective concentration) was reported to range from 0.003 to 0.031 µg mm^−3^ of the liquid culture medium against *Cercospora zeae-maydis* and from 0.12 to 297.22 µg mm^−3^ of the liquid culture medium against *Aspergillus flavus* [10]. Azoxystrobin has often been detected in different ecosystems at higher than acceptable concentrations and therefore may pose a severe threat to organisms found therein [12,13,14].

Pesticides are effective in protecting plants and boosting their yield. However, when used in non-observance of good agricultural practice, they may elicit adverse effects on animal health, as well as water and food quality [15]. Improper and long-term use of fungicides can lead to changes in soil ecosystems as they disturb the abundance, activity, and functioning of the soil microbiota, and in biogeochemical cycles of nitrogen, carbon, phosphorus, and sulfur. This, in turn, may deteriorate the quality and fertility of the soil, which plays a huge role in the environment, by, for instance, providing many nutrients to organisms, increasing plant production, or maintaining biodiversity of the environment [16,17]. Soil organisms contribute to its proper functioning by influencing its physicochemical and biological properties, which ultimately affect crop productivity. Soil microorganisms are essential for maintaining proper ecological balance, soil fertility, plant growth, and pesticide degradation [18]. By secreting various types of enzymes, lipids, and other biologically active macromolecules, they can affect the fate of pesticides in the soil environment [19,20]. Therefore, the assessment of the impact of pesticides, including fungicides, on soil microbiota and soil biochemical processes is a sound action taken to maintain the sustainable development of soil ecosystems [21]. Fungicides and their metabolites can be a major stressor for soil microorganisms, which can lead to both a reduction in their diversity and function in the soil, ultimately affecting ecological functionality [22,23]. Continuous use of fungicides also has detrimental effects on microbial metabolism, soil nutrient cycling, and plant function. Thus, excessive use of these chemicals can lead to high accumulation and prolonged persistence in the soil system, which adversely affects the soil environment [24,25,26]. An example is the study by Verdenelli et al. [27], who noted a significant reduction in the abundance and diversity of gram-positive and gram-negative bacteria and arbuscular fungi under the influence of carbendazim and iprodione applied at the highest dose (4.50 mg kg^−1^ d.m. soil and 8.30 mg kg^−1^ d.m. soil, respectively). Another example is difenoconazole introduced into the soil at 0.04 mg kg^−1^ d.m. soil, which led to a decrease in microbial biomass in loamy-sandy soil, as the microorganisms used more energy to detoxify the environment than for their growth [28]. According to Chamberlain et al. [29], the composition and diversity of microorganisms in the soil affect the regulation of the main functions of the soil, i.e., the circulation of elements, and these functions in turn indirectly affect the growth and yield of plants by, among other things, providing them with nutrients.

Soil enzymes are believed to originate mainly from microorganisms, but also from plant and animal remains entering the soil. They accumulate in the soil as free enzymes or are stabilized on soil organic matter. Soil enzymes are essential for microbial life functions, as they participate in all biochemical processes occurring in the soil, as well as increase the rate of organic matter decomposition reactions, resulting in the release of nutrients into the soil environment. Due to their stability and sensitivity, they are used as indicators of soil health [30]. A study by Filimon et al. [31] showed that difenoconazole introduced into chernozem soil at doses of 37, 75, and 150 mg kg^−1^ d.m. soil under both field and laboratory conditions of all enzymes tested (dehydrogenases, urease, protease, and acid phosphatase) inhibited enzyme activity, with the most inhibitory effect on dehydrogenase activity. The application of another fungicide myclobutanil at a dose of 0.1 mg kg^−1^ d.m. soil contributed to an increase in dehydrogenase activity, while already higher doses (1.0 and 10 mg kg^−1^) significantly inhibited the activity of these enzymes [32]. In a study conducted by Satapute et al. [33] determining the effect of propiconazole (doses of 1.0, 15.0, and 20 kg ha^−1^) on enzymes in red sandy-loam black-earth soil, it was found that the tested fungicide stimulated the activity of urease and phosphatases in the first 2 weeks, while after 3 weeks the activity of these enzymes significantly decreased in sites containing doses of 15.0 and 20.0 mg kg^−1^ of propiconazole. However, the activity of these enzymes was higher in deep-black soil than in red sandy loam.

Fungicides can also be taken up by plants, which can lead to an increase in the production of reactive oxygen species, with subsequent inhibition of normal physiological and biochemical processes in plants and disruption of photosynthesis, thereby reducing yields. Examples of the adverse effects of these chemicals on plants include, for example, that of pendimethalin, which significantly inhibits the germination of *Zea mays* L. seeds as its concentration in the soil increases, or that of fipronil, which significantly reduced the germination of rice seeds compared to control soil [34]. The use of fungicides can cause biochemical and physiological changes in antioxidants, which has an initial effect on plant germination, subsequent growth and development, and ultimately on yield [35].

However, the inactivation of fungicides and their elimination from the soil environment is carried out mainly through microbial processes, as microorganisms are capable of producing enzymes that carry out catabolic processes [36]. It was observed that microorganisms belonging to the Actinomycetota, Proteobacteria, Bacteroidetes, Cyanobacteria, Firmicutes, and Basidiomycota phylum are more abundant in soil contaminated with fungicides than in non-contaminated soil (control soil). Tremendous abilities to degrade fungicides are demonstrated by microorganisms of the genera: *Acinetobacter*, *Achromobacter*, *Agrobacterium*, *Alcaligenes*, *Arthrobacter*, *Azospirillum*, *Enterobacter*, *Bacillus*, *Burkholderia*, *Cupriavidus*, *Flavimonas*, *Brevibacterium*, *Flavobacterium*, *Klebsiella*, *Micrococcus*, *Methylobacterium*, *Mesorhizobium*, *Ochrobactrum*, *Peanibacillus*, *Pseudomonas*, *Pseudaminobacter, Rhizobium*, *Ralstonia*, *Serratia*, *Shinella*, *Sphingomonas Streptomyces*, *Xanthomonas*, and *Yersinia* [37,38].

The aim of this study was, therefore, to assess the effect of soil amendment with two doses of azoxystrobin (field and contaminating doses) on microbiota, enzymes, and plants 30, 60, and 90 days after their application. The study results will allow for a broader understanding of changes in soil microbial populations and biochemical processes taking place in the soil environment. In addition, they may indicate differences in the sensitivity of microorganisms, enzymes, and plants to azoxystrobin. The research hypotheses formulated have assumed that the accumulation of azoxystrobin in soil causes (a) severe disorders in the microbiome, (b) destabilization of enzyme activity, and (c) inhibition of plant growth and development.

## 2. Results

### 2.1. Response of Soil Microbiota to Azoxystrobin

**Figure 1:**
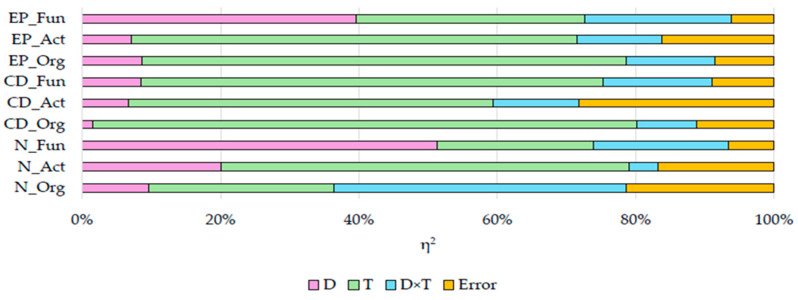
The effects of independent variables (η^2^) on soil microbiological parameters. D—dose of azoxystrobin; T—soil incubation time; D×T—interaction of factors; Org—organotrophic bacteria; Act—actinobacteria; Fun—fungi; N—microbial number; CD—colony development index; EP—ecophysiological diversity index.

Statistical analysis showed that the number of organotrophic bacteria was most affected by the interaction of the studied factors (η^2^ = 42.28%); that of actinobacteria, by soil incubation time (η^2^ = 59.00%); and that of fungi, by azoxystrobin doses (η^2^ = 51.30%) (Figure 1).

Compared to the control soil (soil C), the number of organotrophic bacteria in soil F (field dose) increased 1.2-fold and 1.3-fold on days 30 and 90 of the experiment, respectively, and that of actinobacteria increased 1.9-fold, 1.1-fold, and 1.2-fold on days 30, 60, and 90, respectively, whereas a 1.2-fold decrease was noted in the number of fungi on day 30, a 2.2-fold decrease on day 60, and a 1.4-fold decrease on day 90 (Table 1). In the case of soil P (polluting dose), on day 30 of the experiment, the numbers of organotrophic bacteria and actinobacteria were 1.6-fold and 1.3-fold higher compared to the control, respectively. Analyses conducted on days 60 and 90 demonstrate the inhibition of soil microbiota proliferation in this soil, as evidenced by a 1.3-fold and 1.2-fold decrease in the abundance of organotrophic bacteria, respectively, and a 1.1-fold decrease in that of actinobacteria on both dates. Fungi responded to the highest dose of azoxystrobin with a decrease in their numbers at all dates of analyses. And so, a 1.6-fold decrease was noted in their number in soil P on day 30, a 4.0-fold decrease on day 60, and a 2.6-fold decrease on day 90, compared to the control soil. Regardless of azoxystrobin dose, the highest mean number of organotrophic bacteria (3.069 × 10^9^ cfu kg^−1^ d.m. soil) and actinobacteria (2.105 × 10^9^ cfu kg^−1^ d.m. soil) was observed on day 90, whereas that of fungi was on day 30 (1.350 × 10^7^ cfu kg^−1^ d.m. soil). The most intensive proliferation in the soil was observed in the case of organotrophic bacteria and was the least intensive one—in the case of fungi.

Diversified effects of azoxystrobin over time are confirmed by changes in the population numbers of microorganisms (Table 2). The inhibition in the counts of organotrophic bacteria ranged from 9.50% (soil F, day 60) to 37.98% (soil P, day 60), in those of actinobacteria from 10.46% (soil P, day 60) to 11.66% (soil P, day 90), and in those of fungi from 14.11% (soil F, day 30) to 74.82% (soil P, day 60 day). In the F and P soil samples, the growth of organotrophic bacteria was stimulated on day 30 and inhibited on day 60. In the case of actinobacteria, an increase in their number was recorded at all test dates in soil F, whereas there was a decrease in soil P on days 60 and 90. In turn, the number of fungi decreased in soil F and P in all terms of analyses (days 30, 60, and 90 of the experiment).

**Figure 2:**
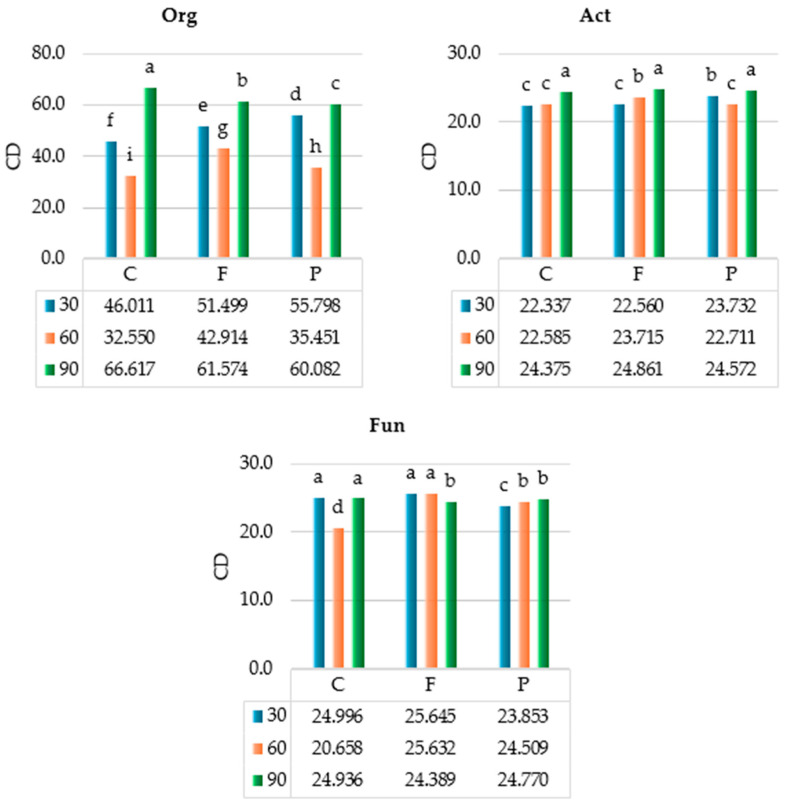
Colony development index (CD) of microorganisms in azoxystrobin-treated soil. C—control soil; F—field dose; P—polluting dose; Org—organotrophic bacteria; Act—actinobacteria; Fun—fungi; 30—30 days of soil incubation; 60—60 days of soil incubation; 90—90 days of soil incubation. Homogeneous groups denoted by letters (^a–i^) were calculated separately for each group of microorganisms (two-way ANOVA analysis performed by Tukey’s method at *p* ≤ 0.01).

The colony development index (CD) of microorganisms was affected to the greatest extent (Figure 2) by the incubation time of soil (from 52.75% to 78.63%), to a lesser extent by the interaction of factors (from 8.58% to 12.39%), and to the least extent by azoxystrobin dose (from 1.54% to 11.79%). Taking into account the incubation time of the soil, the highest CD values of organotrophic bacteria (mean CD = 62.758) and actinobacteria (mean CD = 24.623) were recorded on day 90, and the highest CD of fungi (mean CD = 24.832) on day 30. The CD of organotrophic bacteria was the highest in soil C on day 90 (CD = 66.617), that of actinobacteria in soil F on day 90 (CD = 24.861), and that of fungi in soil F on day 30 (CD = 25.645). Of all groups of microorganisms, the highest CD value was computed for organotrophic bacteria, and the lowest one for fungi.

**Figure 3:**
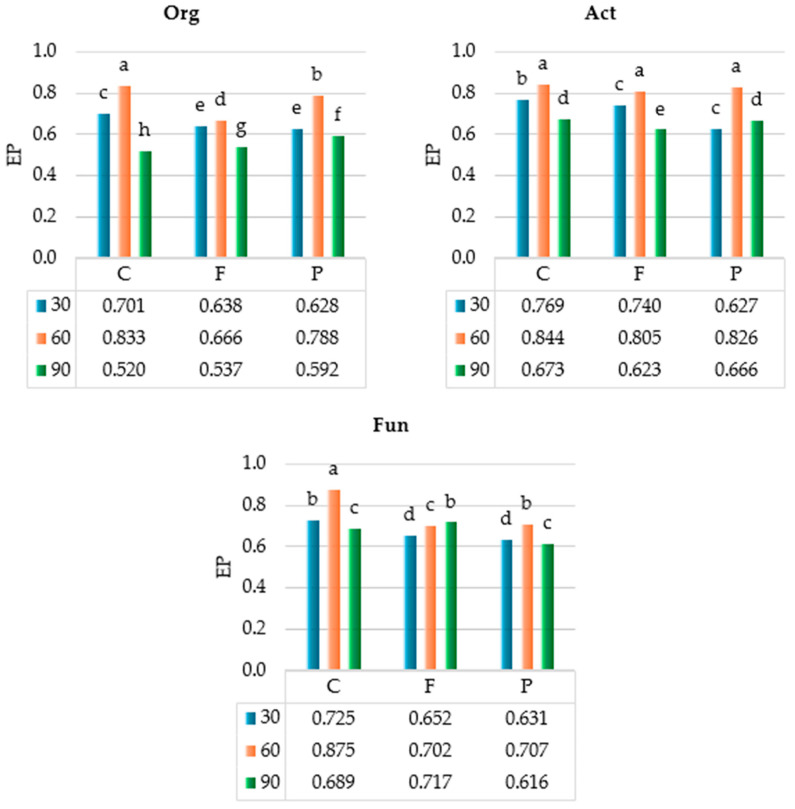
Ecophysiological diversity index (EP) of microorganisms in azoxystrobin-treated soil. C—control soil; F—field dose; P—polluting dose; Org—organotrophic bacteria, Act—actinobacteria, Fun—fungi; 30—30 days of soil incubation; 60—60 days of soil incubation; 90—90 days of soil incubation. Homogeneous groups denoted by letters (^a–h^) were calculated separately for each group of microorganisms (two-way ANOVA analysis performed by Tukey’s method at *p* ≤ 0.01).

Statistical analysis of the observed variance (Figure 3) showed that the incubation time of soil had the strongest impact on the ecophysiological diversity index (EP) of organotrophic bacteria (η^2^ = 64.3%) and actinobacteria (η^2^ = 70.10%), whereas the azoxystrobin dose on EP of fungi was (η^2^ = 51.62%). The organotrophic bacteria, actinobacteria, and fungi had the highest EP values in soil C on day 60 (i.e., 0.833, 0.844, and 0.875, respectively). Regardless of azoxystrobin dose applied, the highest EP values were computed for organotrophic bacteria (mean EP = 0.762), actinobacteria (mean EP = 0.825), and fungi (mean EP = 0.761) on day 60 of the experiment.

Azoxystrobin also contributed to the soil imbalance, as evidenced by the index of soil return to the equilibrium state—resilience index (RL) (Table 3). The greatest changes occurred in the fungal population, because the RL values computed on days 60 and 90 were negative (mean RL values were RL = −0.734 and RL = −0.471, respectively). In the case of organotrophic bacteria and actinobacteria, the mean RL values were positive in all terms of analyses.

**Figure 4:**
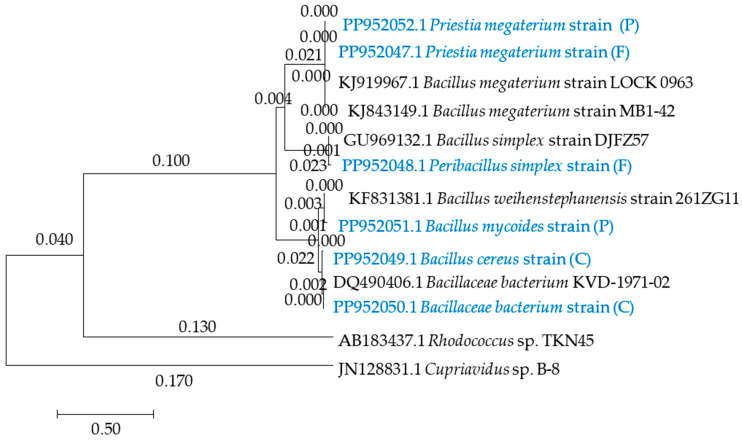
Phylogenetic tree constructed by the neighbor-joining method based on 16S rRNA gene sequences of bacteria isolated from soil at day 90. Bootstrap values expressed as percentages of 500 replications are given at branch points. Bar, 0.50 substitutions per nucleotide position. C—control soil; F—field dose; P—polluting dose. Proprietary bacterial isolates are indicated in blue color, while resistant isolates from the GenBank database are indicated in black color.

**Figure 5:**
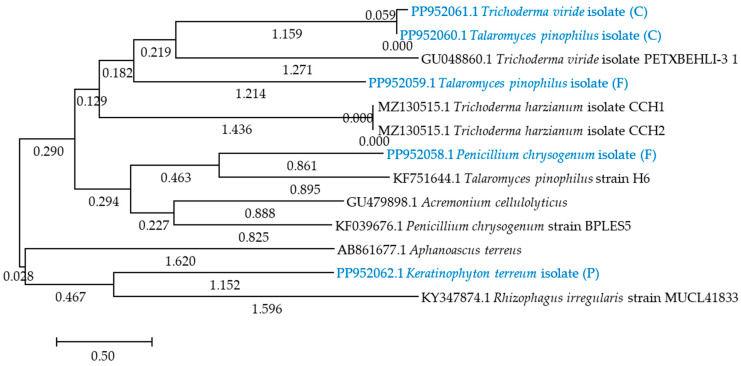
Phylogenetic tree of fungal strains, prepared based on the comparison of the ITS region sequence by the neighbor-joining method of fungi isolated from soil at day 90. Bootstrap values expressed as percentages of 500 replications are given at branch points. Bar, 0.50 substitutions per nucleotide position. C—control soil; F—field dose; P—polluting dose. Proprietary fungal isolates are indicated in blue color, while resistant isolates from the GenBank database are indicated in black color.

Figure 4 shows a phylogenetic tree of bacteria, and Figure 5 shows a phylogenetic tree of fungi. Soil C was most heavily populated by PP952050.1 *Bacillaceae bacterium* strain (C), PP952049.1 *Bacillus cereus* strain (C); and by PP952060.1 *Talaromyces pinophilus* isolate KF751644.1 PP955260.1; and PP952061.1 *Trichoderma pnophilus* isolate (C) fungi. In turn, soil F was colonized by PP952047.1 *Prestia megaterium* strain (F), PP952048.1 *Peribacillus simplex* (F), PP952058.1 *Penicillium chrysogenum* isolate (F), and PP952059.1 *Talaromyces piinophilus* isolate (F), whereas soil P was colonized by PP952052.1 *Prestia megaterium* strain (P), PP952051.1 *Bacillus mycoides* strain (P), and PP952062.1 *Keratinophyton terreum* isolate (P), which may tolerate and degrade azoxystrobin.

### 2.2. Response of Soil Enzymes to Azoxystrobin

**Figure 6:**
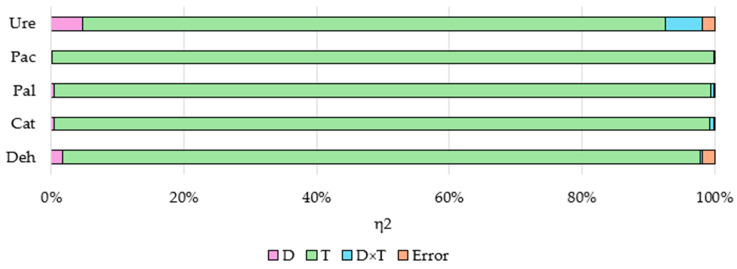
The effects of independent variables (η^2^) on soil enzymatic activity. D—dose of azoxystrobin; T—soil incubation time; D×T—interaction of factors; Deh—dehydrogenases; Cat—catalase; Pal—alkaline phosphatase; Pac—acid phosphatase; Ure—urease.

In this study, soil incubation time had the greatest impact on soil enzyme activity (η^2^ ranged from 87.86% to 99.76%), while the other variables analyzed, namely azoxystrobin dose and interaction of factors, had little effect on soil enzymes (Figure 6).

Regardless of the fungicide dose, the highest dehydrogenases activity (mean 28.361 µmol TPF kg^−1^ d.m. soil h^−1^) and urease activity (mean 2.129 mmol N-NH_4_ kg^−1^ d.m. soil h^−1^) were determined on day 30, whereas the highest catalase activity (mean 0.536 mol O_2_ kg^−1^ d.m. soil h^−1^) and alkaline phosphatase activity (mean 2.369 mmol PNP kg^−1^ d.m. soil h^−1^) were determined on day 60, and the highest acidic phosphatase activity (mean 1.969 mmol PNP kg^−1^ d.m. soil h^−1^) was determined on day 90. In soil F, an increase was observed in all terms of analyses (30, 60, and 90 days) in the activity of dehydrogenases, catalase, and alkaline phosphatase compared to soil C, whereas on day 30 there was an increase in alkaline phosphatase activity, and on day 90 there was a decrease in urease activity. In soil P, dehydrogenases activity decreased compared to soil C in all terms of analyses, alkaline phosphatase and acid phosphatase activities on days 30 and 90, and urease activity on day 90 of the experiment. In the same soil, an increase in catalase activity was observed on days 30 and 60, and an increase in alkaline phosphatase on day 60 (Table 4).

Dehydrogenases activity decreased by 0.45% (soil P, day 90) to 3.75% (soil P, day 60), catalase activity by 1.36% (soil P, day 90), alkaline phosphatase activity by 5.39% (soil P, day 90) to 11.45% (soil P day 30), acid phosphatase activity by 0.98% (soil F, day 90) to 8.55% (soil P, day 90), and urease activity by 0.84% (soil P, day 30) to 18.88% (soil P, day 90) (Table 5). In all analytical terms, the activity of dehydrogenases, catalase, and alkaline phosphatase was stimulated in soil F, whereas dehydrogenases and urease activity were inhibited in soil P. In addition, in soil P, the activity of catalase increased significantly on days 30 and 60, whereas activities of alkaline phosphatase and acid phosphatase were suppressed on days 30 and 90.

Azoxystrobin caused significant changes in sandy clay, which is confirmed by the values of the soil resilience index (Table 6). The greatest disturbance in the soil was noted based on dehydrogenases activity (mean RL = −0.144 on day 60 and RL = −0.037 on day 90) and acid phosphatase activity (mean RL = −0.594 on day 60 and RL = −0.461 on day 90). Adverse changes were also observed in urease activity on day 60 (mean RL = −0.544). The highest mean RL values were determined for catalase activity followed by alkaline phosphatase activity. Their positive RL values indicate that these enzymes are able to return to a state of biochemical equilibrium.

### 2.3. Pearson’s Simple Correlation Coefficients between Microbiological and Biochemical Soil Parameters

**Figure 7:**
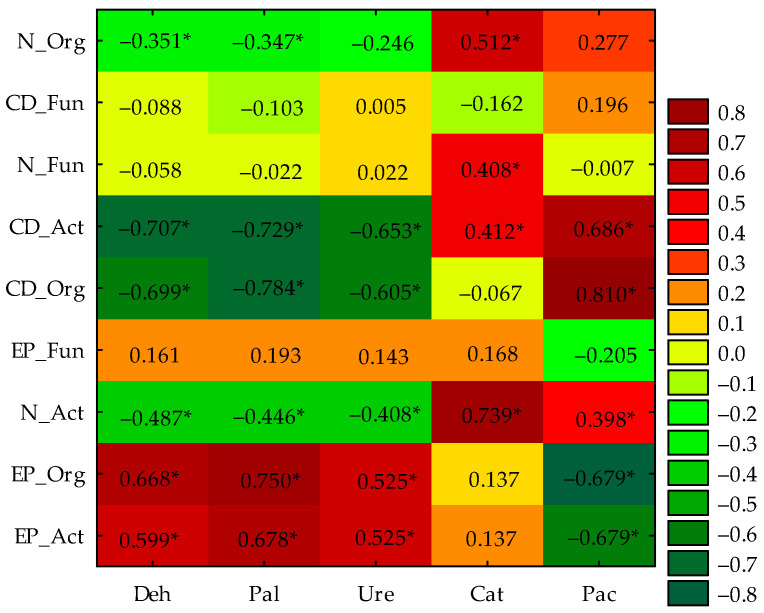
Pearson’s simple correlation coefficients between microbiological and enzymatic properties of soil, *n* = 81; *—significance at *p* = 0.01; N—microbial number; CD—colony development index; EP—ecophysiological diversity index; Org—organotrophic bacteria; Act—actinobacteria; Fun—fungi; Deh—dehydrogenases; Cat—catalase; Pal—alkaline phosphatase; Pac—acid phosphatase; Ure—urease.

The activity of dehydrogenases, alkaline phosphatase, and urease were significantly positively correlated with the EP of organotrophic bacteria and actinobacteria, while negatively correlated with the number of actinobacteria and CD of organotrophic bacteria and actinobacteria. An opposite correlation was found for acid phosphatase. Catalase activity was significantly positively correlated with the abundance of organotrophic bacteria, fungi, and actinobacteria and with the CD of actinobacteria. In addition, the activities of dehydrogenases and alkaline phosphatase were significantly negatively correlated with the count of organotrophic bacteria (Figure 7).

### 2.4. Response of Plants to Azoxystrobin

**Figure 8:**
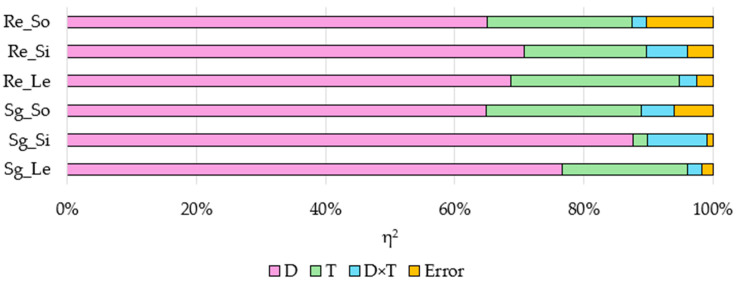
The effects of independent variables (η^2^) on seed germination and root elongation. D—dose of azoxystrobin; T—soil incubation time; D×T—interaction of factors; Sg—seed germination; Re—root elongation; Le—*Lepidium sativum* L.; Si—*Sinapsis alba* L.; So—*Sorgum saccharatum* L.

The percentage of the observed variability (η^2^) of the factors examined showed that the azoxystrobin dose elicited the greatest changes in plant growth and development (Figure 8). Its dose affected seed germination in 64.89% (*S. saccharatum* L.) to 87.57% (*S. alba* L.), while it affected root growth in 65.01% (*S. saccharatum* L.) to 70.68% (*S. alba* L.). The incubation time of the soil modified the seed germination in 2.23% (*S. alba* L.) to 23.97% (*S. saccharatum* L.) and the root growth in 18.95% (*S. alba* L.) to 26.14% (*L. sativum* L.).

The dose of azoxystrobin and duration of its retention in the soil significantly affected the germination of seeds and the elongation of plant roots (Table 7). In soil P, the greatest inhibition of the germination of *L. sativum L.* and *S. saccharatum* L. seeds occurred on day 90 (by 58.43% and 54.23%, respectively) and of *S. alba* seeds on day 60 (by 63.92%). The greatest inhibition of root elongation of plants was recorded on day 90. Compared to the control soil, the root elongation decreased by 54.90% for *L. sativum* L., by 50.92% for *S. alba* L., and by 53.78% for *S. saccharatum* L. A significant inhibition of seed germination and extension of plant roots compared to soil C was also observed in the soil F; however, this inhibition was still less than in the soil P.

## 3. Discussion

### 3.1. Response of Soil Microbiota to Azoxystrobin

Pesticides, including fungicides and their metabolites, can exert an immediate effect on soil microorganisms, triggering changes in their population and diversity [39,40,41]. One such pesticide is azoxystrobin, which, 21 and 28 days after application to the soil in a dose of 10 mg kg^−1^, significantly reduced the numbers of bacteria and actinobacteria compared to the control soil but had no significant effect on the fungi population [42]. In the present study, azoxystrobin applied in the field dose stimulated the growth of organotrophic bacteria and actinobacteria, but it inhibited the growth of fungi. In turn, its contaminating dose reduced the population numbers of all analyzed groups of microorganisms. A small amount of the fungicide in the soil could have been used by organotrophic bacteria and actinobacteria as a source of nutrients; therefore, the recommended dose of azoxystrobin could increase their numbers [43]. However, a fungicide dose being few or several times higher than the agronomic dose may directly affect the survival of microorganisms by disrupting the metabolic pathways in their cells [44]. The sensitivity of microorganisms to increased amounts of azoxystrobin may be due to oxidative stress generated upon the release of electrons from the respiratory chain in the form of reactive oxygen [45].

Fungicides affect not only the proliferation of microbial populations but also their diversity [46,47,48]. They modify the composition of the microbial community because of their effects on non-target organisms [49]. The present study demonstrated differences in the colony development index (CD) and the ecophysiological diversity index (EP) of microorganisms. In the soil treated with azoxystrobin, there was an increase in the CD values computed for organotrophic bacteria, actinobacteria, and fungi compared to the control soil. The r-strategies (fast-growing microorganisms) prevailed among organotrophic bacteria, whereas the k-strategists (slow-growing microorganisms) prevailed among actinobacteria and fungi. This was evidenced by the CDs, the mean values of which were CD = 50.27 for organotrophic bacteria and CD = 23.49 and CD = 24.38 for actinobacteria and fungi, respectively. Therefore, it can be concluded that the addition of chemical compounds to the soil may determine the proportions between r-strategists and k-strategists [50,51,52]. Azoxystrobin exerted diverse effects on the EP of microorganisms, which was also caused by the time of its retention in the soil. However, the greatest changes caused by azoxystrobin presence in the soil, which were manifested by EP decrease, were observed in the case of fungi. The adaptation of microorganisms to adverse conditions is largely dependent on their activity, as well as on the degree of severity of the stress factor occurring in the soil environment [51,52].

Fungicides are degraded in the soil environment by various microorganisms that produce specific enzymes capable of their degradation [53,54]. For example, Alexandrino et al. [39] demonstrated that bacteria of the genera *Pseudomonas*, *Rhodobacter, Ochobacterum*, *Comamonas*, *Hydrogenophaga*, *Azospirillum*, *Methylbacillus*, and *Acinetobacter* had high degradation potential against epoxiconazole and fludioxonil, as they degraded 10 mg dm^−3^ of these fungicides within 21 days. In turn, Feng et al. [8] have reported that *Arthrobacter*, *Bacillus*, *Cupriavidus*, *Pseudomonas*, *Klebsiella*, *Rhodanobacter*, *Stenothrofomonas*, and *Aphanoascus* are microorganisms that break down strobilurin compounds. Clinton et al. [55] isolated two species of bacteria from the soil contaminated with trifloxystrobin, namely *Bacillus flexus* and *Bacillus amyloliquefaciens*, whereas Howell et al. [56] observed that *Cuprividus* spp. and *Rhodobacter* spp. exerted a degrading effect against azoxystrobin. *Actinomyces* spp. and *Ochrabactrum* spp. are also capable of degrading azoxystrobin [57]. In our study, we identified microorganisms, i.e., bacteria PP952052.1 *Prestia megaterium* strain (P) and PP952051.1 *Bacillus mycoides* strain (P), and fungi PP952062.1 *Keratinophyton terreum* isolate (P), which may show high tolerance to azoxystrobin and the potential for its degradation. In turn, Feng et al. [58] isolated a strain of bacteria *Chrobacrum anthropi* SH14 from soil contaminated with azoxystrobin, which was able to degrade 86.30% of the 50 µg cm^−3^ medium dose of this pesticide within 5 days.

### 3.2. Response of Soil Enzymes to Azoxystrobin

The activity of soil enzymes is closely related to the quality and fertility of the soil. These biological parameters of the soil respond quickly to the effects of high pesticide doses [59]. Wang et al. [42] reported that azoxystrobin, used at doses of 0.1 to 10 mg kg^−1^, inhibited dehydrogenases activity in all terms of analyses (i.e., on days 7, 14, 21, and 28) and urease activity up to day 14 while stimulating catalase activity and not significantly affecting protease activity. In the experiment described in this manuscript, the contaminating dose of azoxystrobin inhibited the activity of dehydrogenases, alkaline phosphatase, acid phosphatase, and urease, while its agronomic dose enhanced activities of all analyzed soil enzymes. The inactivating effect of azoxystrobin on soil enzymes may have been due to the inhibition of microbial population multiplication, which indirectly affected the secretion of enzymes whose activity is strongly dependent on the number and biomass of microorganisms [60]. Adverse effects of azoxystrobin (doses: 2.90, 14.65, and 35.00 mg kg^−1^) on the activity of soil enzymes such as dehydrogenases, urease, alkaline phosphatase, acid phosphatase, arylsulfatase, and *β*-glucosidase were noted in the study by Boteva et al. [61], with dehydrogenases and arylsulfatase being the most sensitive, and urease being the most resistant to soil treatment with this pesticide. In the present study, catalase was the most resistant to azoxystrobin, as evidenced by its enhanced activity in the soil contaminated with this compound. The increase in its activity may suggest that some microorganisms used azoxystrobin as a source of nutrients and energy necessary for their growth, which contributed to boosted catalase secretion by their cells [62]. The increased catalase production by microorganisms probably caused fungicide degradation and strengthened the protective barrier of microorganisms against oxidizing compounds [60]. The positive or negative effects of azoxystrobin on soil enzymes are mainly related to its dose and duration of its retention in the soil [59].

### 3.3. Response of Plants to Azoxystrobin

In addition to their antifungal activity, strobilurins improve plant quality by intensifying photosynthesis; increasing contents of nitrogen, chlorophyll, and protein; and delaying the aging of plants [45]. Amaro et al. [63] and Chiu-Yueh et al. [64] have pointed to a very strong effect of fungicides from the group of strobilurin compounds on the physiology and growth of plants. In the present study, azoxystrobin added to soil both in the recommended field dose and the contaminating dose, inhibited seed germination and elongation *of L. sativum* L., *S. alba* L., and *S. saccharatum* L. shoots in all analytical terms (days 30, 60, and 90). Eman et al. [65] determined the effect of azoxystrobin applied at the recommended dose and also at doses 0.5-fold and 2-fold higher than the recommended dose on the germination of *Triticum aestivum* L. and *Raphanus sativus* L. seeds. They found that azoxystrobin significantly reduced their seed germination percentage and the length of their roots and shoots. Particularly significant reduction in the length of roots and shoots *of Triticum aestivum* L. and *Raphanus sativus* L. was reported at a 2-fold-higher dose than the recommended one, which reduced the length of roots and shoots in wheat by 13.20% and 26.02%, respectively and in radish by 17.67% and 51.67%, respectively. In the present study, an azoxystrobin dose of 32.92 mg kg^−1^ caused, on average, 50.31% and 45.26% reduction in the length of shoots and roots of *L. sativum* L., respectively; 57.52% and 48.29% reductions in *S. alba* L.; and 45.32% and 44.65% reductions in these traits in *S. saccharatum* L., respectively. The impaired plant growth could have been due to the blocking of the cytochrome bc1 complex, which inhibited cell division and water uptake by plants [66]. Amaro et al. [63], who assessed the effect of an azoxystrobin dose of 60 g ha^−1^, found a reduction in the rate of CO_2_ assimilation, transpiration, stomata conductivity, and carbon concentration in cucumber plants.

## 4. Materials and Methods

### 4.1. Soil Materials

Soil material was collected from the humus-horizontal soil depth of 0 to 20 cm from Tomaszkowo, located in the north-eastern part of Poland (53.71610° N, 20.41670° E). This was soil belonging to the Eutric Cambisols subtype, which was formed on sandy loam (69.41% sand fraction, 27.71% clay fraction, and 2.88% silt fraction) [67]. Selected physicochemical and chemical properties of the soil (soil granulometric composition; pH, hydrolytic acidity; sum of base exchangeable cations bases; organic carbon content; total nitrogen content; and total exchangeable cations K^+^, Na^+^, Ca^2+^, and Mg^2+^) can be found in Table 8. The analyses were performed in 3 replicates according to the methodology described in the study by Borowik et al. [68].

### 4.2. Azoxystrobin

**Figure 9:**
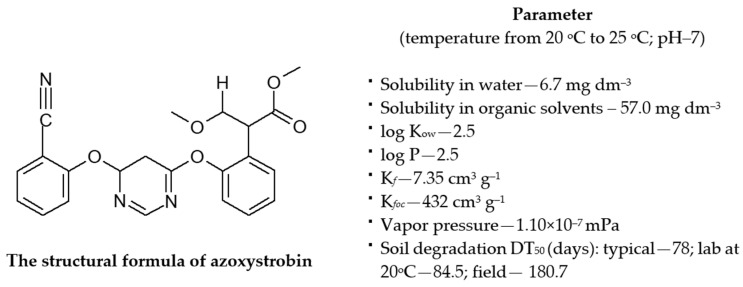
Selected physicochemical properties of azoxystrobin [71].

The experiment conducted introduced azoxystrobin into the soil in the form of Amistar 250 SC (azoxystrobin amounts to 250 g dm^−3^ of the formulation) as a pure substance at rates of 0.110 mg kg^−1^ (field dose) and 32.92 mg kg^−1^ (polluting dose). The formulation was manufactured by Syngenta Crop Protection AG (Stein, Switzerland). The formulation was marketed in Poland in 2011, and the distribution authorization was granted to Syngenta (Warsaw, Poland). The expiry date of the authorization of the preparation of Amistar 250 SC by the company Syngenta is 31 December 2025 [69]. The single dose recommended by the manufacturer amounts to 0.5 to 3.0 dm^3^ ha^−1^. This preparation is used in the protection of crops (winter wheat, spring wheat, rye, winter barley, spring barley, winter triticale, spring triticale, and winter oilseed rape) and vegetable crops (potato, onion, green bean, green pea, head cabbage, Chinese cabbage, cauliflower, carrot, lettuce, tomato, leek, celery, and pepper). The selected physicochemical properties of azoxystrobin are presented in Figure 9. The structural formula of azoxystrobin was made using ISIS Draw 2.3 [70].

### 4.3. Establishment of the Experiment and Procedure for Conducting the Experiment

The procedure for setting up an experiment under strictly controlled conditions (laboratory experiment) in 3 replicates for each combination and each test date (27 beakers in total). The procedure for setting up the experiment consisted of weighing 100 g each of air-dried soil put through a sieve (2 mm diameter) into glass beakers (150 cm^3^ capacity). In the respective sites, azoxystrobin in the form of an aqueous emulsion was applied once in the following amounts (mg kg^−1^ d.m. soil): 0.00 mg (soil without added fungicide), 0.110 mg (field dose), and 32.92 mg (polluting dose). The literature generally describes studies on the impact of small doses of azoxystrobin on soil properties and plant development [2,8,43,45,60]. Therefore, our research aimed to assess the impact of this active substance in contaminating amounts on the biological parameters of the soil. The soil material was thoroughly homogenized and brought to a moisture content of 50% of the capillary water capacity using distilled water. The soil in the beaker was covered with perforated foil and incubated in a thermostat maintaining a constant temperature (25 °C) for 30, 60, and 90 days. Soil moisture was monitored throughout the experiment, and soil losses were replenished. Soil microbiological and enzymatic analyses were performed on three test dates. For the Phytotoxkit tests, a separate batch of the experiment was set up (9 replicates for each combination and each test date, resulting in a total of 81 beakers). A total of 150 g of soil was weighed into each beaker. The conditions for setting up and running the experiment were identical to those for the soil used for microbiological and enzymatic analysis.

### 4.4. Conducting Microbiological Analysis of Soil

**Figure 10:**
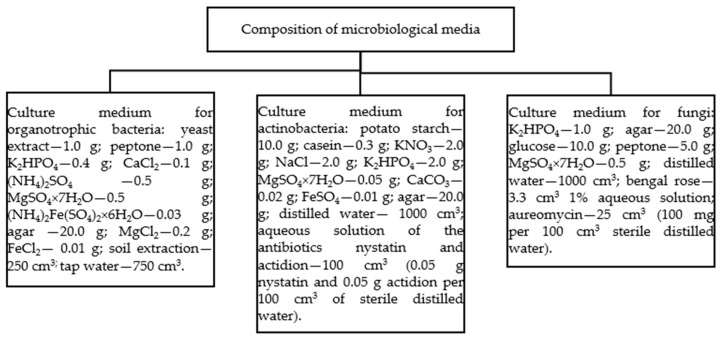
Composition of microbiological media intended for the cultivation of the total number of organotrophic bacteria, actinobacteria and fungi.

At three study dates (30, 60, and 90 days), soil microbiological analysis was carried out using the serial dilution method. Into 90 cm^3^ of sterile saline (0.85% NaCl) were weighed 10 g of soil of each sample analyzed; then, the whole was mixed on a shaker (120 rpm for 30 min), and a series of dilutions were made. An amount of 1 cm^3^ of the specified dilution (organotrophic bacteria and actinobacteria—10^−5^, fungi—10^−3^) and 17 cm^3^ of selective medium were introduced into sterile Petri dishes in parallel. Bunt and Rovira medium for organotrophic bacteria, Küster and Williams medium for actinobacteria, and Martin medium for fungi were used for culture. The microbial material was incubated for 10 days in a thermostat at 28 °C; the grown colonies of microorganisms were counted each day. The composition of the microbial media is presented in Figure 10. The exact procedure for performing the microbiological analysis is described according to Kucharski et al. [72] and Wyszkowska et al. [73]. These analyses were performed in 9 replicates for each combination. Each day, the grown colonies of microorganisms were counted. The number of microorganisms was expressed in colony-forming units per kg of soil (cfu kg^−1^ d.m. soil).

### 4.5. Isolation of Microorganisms from Soil and Their Identification

**Figure 11:**
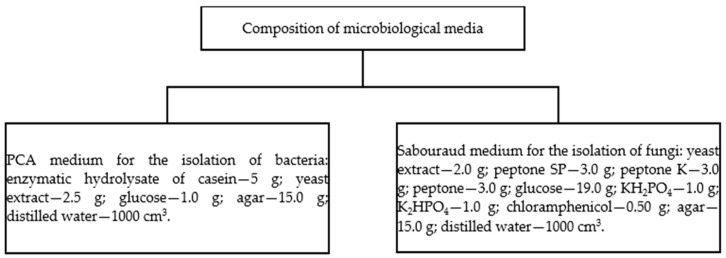
Composition of microbiological media for the isolation of bacteria and fungi.

On day 90 of the experiment, bacteria and fungi were isolated from the control soil and the soil containing azoxystrobin in the amounts of 0.110 mg kg^−1^ and 32.92 mg kg^−1^ by serial dilution. Isolation of bacteria and fungi was carried out by making serial dilutions by suspending 10 g of each of the soil samples analyzed in sterile saline (0.85% NaCl) (1:10 ratio). The prepared dilutions (bacteria—10^−5^ and fungi—by 10^−3^) were introduced at a rate of 1 cm^3^ into a Petri dish (3 repetitions). PCA medium was used to grow bacteria, while fungi were grown in Sabouraud medium, the composition of which is presented in Figure 11. The prepared microbial material was incubated at 37 °C (from 24 to 48 h). Serial passaging of characteristic colonies of microorganisms was performed to obtain pure cultures.

**Figure 12:**
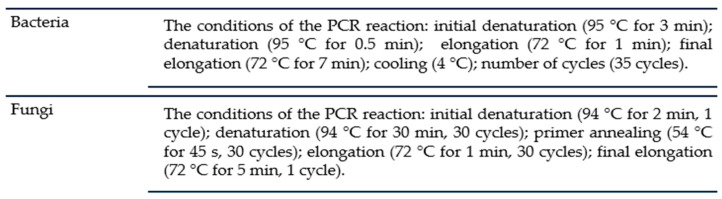
Conditions for conducting the PCR reaction.

Genomic DNA was isolated using a Bead-Beat Micro Gravity kit (A&A Biotechnology, Gdansk, Poland), which separated DNA by electrophoresis in a 1.0% agarose gel (5 mm^3^ sample per gel). For the PCR reaction, a reaction mixture of the following composition was used: 5 mm^3^ (~50 ng) of genomic DNA, 25 mm^3^ of 2× PCR Master Mix Plus High GC (A&A Biotechnology, Gdansk, Poland), 0.2 mm^3^ of each primer at 100 μM, and 19.6 mm^3^ of sterile water. B-all For (GAG TTT GAT CCT GGC TCA G) and B-all Rev (ACG GCT ACC TTA CGA CTT) primers were used to isolate the 16S rRNA gene of bacteria, while ITS1 (TTC GTA GGT GAA CCT GCG G) and ITS4 (TCC TCC GCT TAT TGA TAT GC) primers were used to isolate the ITS region of fungi. Conditions for the PCR reaction can be found in Figure 12.

After the PCR reaction on an agarose gel (2.0%), the reaction mixture was separated (2 mm^3^ of sample per gel), and the amplified DNA fragments were purified using the Clean-Up AX kit (A&A Biotechnology, Poland). The resulting PCR products were resuspended in 10.0 mM Tris-HCl pH 8.0 and diluted to a concentration of 100 ng mm^−3^. DNA sequencing was performed by Macrogen (Amsterdam, Netherlands) on a 3730 XL Analyzer DNA analyzer (Life Technologies Holding Pte Ltd., Singapore) [52,74,75,76]. The DNA sequences obtained were compared with GenBank (National Center of Biotechnology Information) data. The DNA sequences of the *16S rRNA* subunit of bacteria were compared using BLAST (Basic Local Alignment Search Tool) software [https://blast.ncbi.nlm.nih.gov/Blast.cgi (accessed on 1 July 2024)], while the ITS regions of fungi were compared using Internal Transcribed Spacer software [https://www.applied-maths.com/download/software (accessed on 1 July 2024)]. The access in the GenBank database for the nucleotide sequences of bacteria are under numbers ranging from PP952047 to PP952052 [https://www.ncbi.nlm.nih.gov/nuccore/PP952047.1:PP952052 (accessed on 1 July 2024), https://www.ncbi.nlm.nih.gov/nuccore (accessed on 1 July 2024)], while those of fungi are under numbers ranging from PP952058 to PP952062 [https://www.ncbi.nlm.nih.gov/nuccore/PP952058.1:PP52062.1 (accessed on 1 July 2024)]. Based on the obtained nucleotide sequences of the identified microorganisms, a phylogenetic tree was created using the neighbor-joining method with the MEGA 11 software [https://www.megasoftware.net/show_eua (accessed on 1 July 2024)] [77]. The conditions for creating the phylogenetic tree in MEGA 11 software were as follows: statistical method—neighbor-joining (NJ); scope—all selected taxa; test phylogeny—bootstrap method (no. of bootstrap reconstruction—500 for bacteria and fungi); substitution type—nucleotide; model/methods—Maximum Composite Likelihood; substitution of include—d: transitions and transversion; rates among sites—uniform rates; pattern among lineages—same (homogenous); gaps/missing data treatment—partial deletion; select codon positions—1st + 2nd + 3rd + noncoding; number of threads—1; searching NJ tree—100%, by sequence difference—0.066 for bacteria and 0.036 for fungi.

### 4.6. Conducting Enzymatic Analysis of Soil

Enzymatic analyses of the soil were performed at 30, 60, and 90 days (9 replicates) determining the activity of dehydrogenases, catalase, alkaline phosphatase, acid phosphatase, and urease. Then, 2,3,5-triphenyl tetrazolium chloride was used to determine dehydrogenases activity, hydrogen peroxide—catalase, 4-nitrophenyl phosphate disodium—alkaline phosphatase, acid phosphatase, and urea—urease. Dehydrogenase activity was determined by the Öhlinger method, catalase by the Johnson and Temple method, and alkaline phosphatase and urease by the Alef and Nannpieri method. These analyses were performed according to the procedure given in the studies by Wyszkowska et al. [73] and Wyszkowska et al. [78].

### 4.7. The Effect of Azoxystrobin on Seed Germination and Plant Root Elongation

The effects of azoxystrobin on the growth and development of plants (*Lepidium sativum* L., *Sinapsis alba* L., and *Sorgum saccharatum* L.) were assessed at specific test dates (30, 60, and 90 days) using the Phytotoxkit test. The soil (110 g) from the control (soil without added fungicide) and with azoxystrobin at 0.110 mg kg^−1^ and 32.92 mg kg^−1^ was introduced onto plastic plates. Then, 10 seeds of each plant were placed on moist filter paper in 3 replicates. The material thus prepared was incubated for 72 h (temperature 25 °C). The shoot and root lengths of the test plants were then measured.

### 4.8. Calculation of Results

The following soil biological indices were calculated at each test date (30, 60, and 90 days):

▪ Colony development index (CD) of microorganisms [79]: CD values range from 0 to 100. CD values close to 100 indicate rapid growth of the microorganism population in a short period.

(1) $$CD=\left(\frac{N_{1}}{1}+\frac{N_{2}}{2}+\frac{N_{3}}{3}+\cdots +\frac{N_{10}}{10}\right)\times 100$$

where N_1_, N_2_, N_3_, …, N_10_—is the ratio of the total number of microbial colonies grown (1, 2, 3, …, 10th day of incubation) to the total number of microbial colonies grown over the entire incubation period (10 days);

▪ The ecophysiological diversity index (EP) of microorganisms [80] takes values from 0 to 1, measuring the stability and homogeneity of microorganisms over time. EP values close to 1 indicate steady growth of microorganisms in the environment.

(2) $$EP=-Ʃ(p_{i}\times log10p_{i})$$

where p_i_ is the ratio of the sum of the number of microbial colonies grown per incubation day to the total number of microbial colonies grown over the entire incubation period (10 days);

▪ Changes (Ch_a_) of microbial abundance, enzyme activity, seed germination, and root elongation in soil caused by azoxystrobin: A positive Ch_a_ value indicates stimulation of the analyzed parameters under the influence of azoxystrobin, while a negative value indicates inhibition.

(3) $${Ch}_{a}=\frac{\left(A-C\right)}{C}\times 100\%$$

where A represents values of analyzed parameters in the soil with azoxystrobin, and C represents values of analyzed parameters in the control soil;

▪ The resilience index (RL) of azoxystrobin-treated soil is determined by microbial abundance and enzyme activity [81]. RL values range from −1 to 1. A RL value close to −1 indicates that the soil is not returning to equilibrium. A RL value close to 1 indicates that the soil is returning to equilibrium. A RL value close to 0 indicates that the soil is out of or slightly out of equilibrium.

(4) $${\mathrm{RLat}}_{tx}=\frac{2|D_{0}|}{\left({|C}_{0}|+|D_{x}|\right)}$$

where D_0_ is the difference in soil microbial numbers and enzyme activity between a control soil sample (C_0_) and an azoxystrobin-treated soil sample (t_0_), and D_x_ is the difference in soil microbial numbers and enzyme activity between a control and an azoxystrobin-treated sample after 60 and 90 days of soil incubation.

### 4.9. Statistical Analyses of Results

The results obtained were statistically processed with a two-factor (factor 1: azoxystrobin dose, factor 2: soil incubation time) ANOVA analysis of variance (*p* ≤ 0.01) using Statistica 13.3 software [82]. The percentage of observed variability in the soil parameters studied was determined using the η^2^ coefficient. Homogeneous groups were calculated using Tukey’s test at *p* ≤ 0.01 to determine the most significant differences between mean values. Pearson’s simple correlation coefficients between microbiological and biochemical soil parameters were presented as a heat map.

## 5. Conclusions

Azoxystrobin was observed to induce changes in soil microbiome and enzymatic activity and also in plant growth and development over time. Its field dose (0.110 mg kg^−1^) increased the numbers of organotrophic bacteria and actinobacteria, the CD values, and the activity of soil enzymes. In turn, it reduced the number of fungi, decreased the EP values, and inhibited seed germination and root elongation of the tested plants. In turn, its contaminating dose (32.92 mg kg^−1^) reduced the number of fungi; suppressed activities of dehydrogenases, alkaline phosphatase, acid phosphatase, and urease; and decreased the EP values, while increasing the CD values and enhancing catalase activity. In addition, it significantly inhibited seed germination and root elongation of *Lepidium sativum* L., *Sinapsis alba* L., and *Sorgum saccharatum*. It was observed that control soil (soil not contaminated with the fungicide) was most heavily populated by the genera bacteria PP952050.1 *Bacillaceae bacterium* strain (C), PP952049.1 *Bacillus cereus* strain (C), fungi PP952060.1 *Talaromyces pinophilus* isolate (C), and PP952061.1 *Trichoderma viride* isolate (C), while the contaminated soil was most heavily populated by bacteria PP952052.1 *Prestia megaterium* strain (P), PP952051.1 *Bacillus mycoides* strain (P), and fungi PP952062.1 *Keratinophyton terreum* isolate (P). The effects of azoxystrobin on the microbiota, enzymes, and plants varied over time, depending on dose, the species of microorganisms and plants, and enzyme type. The study results indicate that azoxystrobin can trigger significant changes in soil biological parameters, particularly when applied in the contaminating dose. It caused permanent disorders in the growth of fungi and the activity of dehydrogenases, acid phosphatase, and urease, as evidenced by negative values of the RL. The identification of bacteria and fungi in the soil containing azoxystrobin can be harnessed to restore soils contaminated with this fungicide by their bioaugmentation with resistant and degrading strains.

## Figures and Tables

**Table 1:** Microbial number in azoxystrobin-treated soil, cfu 10^n^ kg^−1^ d.m. soil.

Object	Org			Act			Fun		
	Soil Incubation Time (Days)								
	30	60	90	30	60	90	30	60	90
C	1.720 ^i^	3.378 ^b^	2.940 ^d^	0.832 ^h^	1.865 ^d^	2.059 ^c^	1.651 ^d^	4.798 ^a^	3.193 ^b^
F	2.133 ^g^	3.057 ^c^	3.772 ^a^	1.575 ^f^	2.142 ^b^	2.437 ^a^	1.418 ^e^	2.158 ^c^	2.197 ^c^
P	2.780 ^e^	2.095 ^h^	2.496 ^f^	1.115 ^g^	1.670 ^e^	1.819 ^d^	0.982 ^g^	1.208 ^f^	1.246 ^f^
Average	2.211 ^C^	2.843 ^B^	3.069 ^A^	1.174 ^C^	1.892 ^B^	2.105 ^A^	1.350 ^C^	2.721 ^A^	2.212 ^B^

C—control soil; F—field dose; P—polluting dose; Org—organotrophic bacteria; Act—actinobacteria; Fun—fungi; n was for Org and Act—10^9^, Fun—10^7^. Homogeneous groups denoted by letters (^a–i^) were calculated separately for each group of microorganisms (two-way ANOVA analysis performed by Tukey’s method at *p* ≤ 0.01). Capital letters (^A–C^) denote homogeneous groups for the test dates separately for each group of microorganisms.

**Table 2:** Changes in the numbers of microorganisms (Ch_a_) under the influence of azoxystrobin (%).

Object	Org			Act			Fun		
	Soil Incubation Time (Days)								
	30	60	90	30	60	90	30	60	90
F	24.012 ^c^	−9.503 ^d^	28.299 ^b^	89.303 ^a^	14.852 ^d^	18.358 ^c^	−14.113 ^a^	−55.023 ^d^	−31.193 ^b^
P	61.678 ^a^	−37.981 ^f^	−15.102 ^e^	34.014 ^b^	−10.456 ^e^	−11.656 ^f^	−40.521 ^c^	−74.823 ^f^	−60.977 ^e^
Average	42.845 ^A^	−23.742 ^C^	6.598 ^B^	61.685 ^A^	2.198 ^C^	3.351 ^B^	−27.317 ^A^	−64.923 ^C^	−46.085 ^B^

F—field dose; P—polluting dose; Org—organotrophic bacteria; Act—actinobacteria; Fun—fungi. Homogeneous groups denoted by letters (^a–f^) were calculated separately for each group of microorganisms (two-way ANOVA analysis performed by Tukey’s method at *p* ≤ 0.01). Capital letters (^A–C^) denote homogeneous groups for the test dates separately for each group of microorganisms.

**Table 3:** Resilience index (RL) for azoxystrobin-treated soil based on microbial number.

Object	Org		Act		Fun	
	Soil Incubation Time (Days)					
	60	90	60	90	60	90
F	0.131 ^b^	−0.288 ^d^	0.451 ^a^	0.038 ^c^	−0.779 ^d^	−0.450 ^a^
P	−0.089 ^c^	0.367 ^a^	0.123 ^b^	−0.038 ^d^	−0.689 ^c^	−0.491 ^b^
Average	0.021 ^B^	0.040 ^A^	0.287 ^A^	0.000 ^B^	−0.734 ^B^	−0.471 ^A^

F—field dose; P—polluting dose; Org—organotrophic bacteria; Act—actinobacteria; Fun—fungi. Homogeneous groups denoted by letters (^a–d^) were calculated separately for each group of microorganisms (two-way ANOVA analysis performed by Tukey’s method at *p* ≤ 0.01). Capital letters (^A,B^) denote homogeneous groups for the test dates separately for each group of microorganisms.

**Table 4:** Enzymatic activity in azoxystrobin-treated soil, kg^−1^ d.m. soil h^−1^.

Object	Deh (µmol TPF)			Cat (mol O_2_)			Pal (mmol PNP)			Pac (mmol PNP)			Ure (mmol N-NH_4_)		
	Soil Incubation Time (Days)														
	30	60	90	30	60	90	30	60	90	30	60	90	30	60	90
C	28.050 ^a^	27.454 ^b^	19.159 ^e^	0.086 ^e^	0.516 ^b^	0.515 ^b^	2.261 ^f^	2.266 ^f^	0.334 ^b^	1.327 ^c^	1.048 ^e^	2.034 ^a^	2.129 ^a^	2.089 ^b^	1.721 ^c^
F	28.359 ^a^	28.139 ^a^	19.719 ^d^	0.102 ^d^	0.542 ^a^	0.521 ^a^	2.325 ^e^	2.455 ^d^	0.363 ^a^	1.301 ^c^	1.133 ^d^	2.014 ^a^	2.147 ^a^	2.127 ^b^	1.671 ^d^
P	27.034 ^b^	26.424 ^c^	19.073 ^f^	0.156 ^c^	0.549 ^a^	0.508 ^b^	2.002 ^g^	2.387 ^e^	0.316 ^c^	1.220 ^d^	1.126 ^e^	1.860 ^b^	2.111 ^a^	2.078 ^b^	1.396 ^e^
Average	28.361 ^A^	27.339 ^B^	19.317 ^C^	0.115 ^C^	0.536 ^A^	0.515 ^B^	2.196 ^B^	2.369 ^A^	0.338 ^C^	1.283 ^C^	1.102 ^B^	1.969 ^A^	2.129 ^A^	2.098 ^B^	1.596 ^C^

C—control soil; F—field dose; P—polluting dose; Deh—dehydrogenases; Cat—catalase; Pal—alkaline phosphatase; Pac—acid phosphatase; Ure—urease. Homogeneous groups denoted by letters (^a–g^) were calculated separately for each enzyme (two-way ANOVA performed by Tukey’s method at *p* ≤ 0.01). Capital letters (^A–C^) denote homogeneous groups for the test dates separately for each enzyme.

**Table 5:** Changes in the activity enzymes (Ch_a_) of soil under the influence of azoxystrobin (%).

Object	Deh			Cat			Pal			Pac			Ure		
	Soil Incubation Time (Days)														
	30	60	90	30	60	90	30	60	90	30	60	90	30	60	90
F	1.102 ^c^	2.495 ^b^	2.923 ^a^	18.605 ^b^	1.342 ^d^	1.165 ^e^	2.831 ^d^	8.341 ^b^	8.683 ^a^	−1.959 ^d^	8.112 ^a^	−0.983 ^c^	0.845 ^b^	1.819 ^a^	−2.905 ^e^
P	−3.622 ^e^	−3.752 ^f^	−0.449 ^d^	81.395 ^a^	6.395 ^c^	−1.359 ^f^	−11.455 ^f^	5.340 ^c^	−5.389 ^e^	−8.063 ^e^	7.443 ^b^	−8.554 ^f^	−0.845 ^c^	−1.100 ^d^	−18.884 ^f^
Average	−1.260 ^C^	−0.628 ^B^	1.237 ^A^	50.000 ^A^	3.868 ^B^	−0.097 ^C^	−4.312 ^C^	6.840 ^A^	1.647 ^B^	−5.011 ^C^	7.777 ^A^	−4.768 ^B^	0.000 ^B^	0.359 ^A^	−10.894 ^C^

F—field dose; P—polluting dose; Deh—dehydrogenases; Cat—catalase; Pal—alkaline phosphatase; Pac—acid phosphatase; Ure—urease. Homogeneous groups denoted by letters (^a–f^) were calculated separately for each enzyme (two-way ANOVA performed by Tukey’s method at *p* ≤ 0.01). Capital letters (^A–C^) denote homogeneous groups for the test dates separately for each enzyme.

**Table 6:** Resilience index (RL) for azoxystrobin-treated soil based on enzyme activity.

Object	Deh		Cat		Pal		Pac		Ure	
	Soil Incubation Time (Days)									
	60	90	60	90	60	90	60	90	60	90
F	−0.362 ^d^	−0.269 ^c^	−0.241 ^d^	0.467 ^b^	−0.166 ^d^	0.370 ^c^	−0.549 ^c^	−0.499 ^b^	−0.597 ^d^	0.854 ^a^
P	0.075 ^b^	0.195 ^a^	0.359 ^c^	0.843 ^a^	0.783 ^b^	0.876 ^a^	−0.638 ^d^	−0.423 ^a^	−0.491 ^c^	−0.267 ^b^
Average	−0.144 ^B^	−0.037 ^A^	0.059 ^B^	0.655 ^A^	0.309 ^B^	0.623 ^A^	−0.594 ^B^	−0.461 ^A^	−0.544 ^B^	0.294 ^A^

F—field dose; P—polluting dose; Deh—dehydrogenases; Cat—catalase; Pal—alkaline phosphatase; Pac—acid phosphatase; Ure—urease. Homogeneous groups denoted by letters (^a–d^) were calculated separately for each enzyme (two-way ANOVA performed by Tukey’s method at *p* ≤ 0.01). Capital letters (^A,B^) denote homogeneous groups for the test dates separately for each enzyme.

**Table 7:** Changes in seed germination and root elongation (Ch_a_) under the influence of azoxystrobin using the Phytotoxkit test (%).

Object	*Lepidium sativum* L.			*Sinapsis alba* L.			*Sorghum saccharatum* L.		
	Soil Incubation Time (Days)								
	30	60	90	30	60	90	30	60	90
Inhibition of seed germination									
F	−21.847 ^a^	−41.681 ^c^	−38.777 ^b^	−55.130 ^c^	−56.910 ^c^	−22.919 ^a^	−9.202 ^a^	−19.756 ^b^	−44.299 ^e^
P	−40.442 ^c^	−52.044 ^d^	−58.432 ^e^	−56.257 ^c^	−63.924 ^d^	−52.375 ^b^	−39.323 ^c^	−42.413 ^d^	−54.227 ^f^
Average	−31.145	−46.863	−48.605	−55.693	−60.417	−37.647	−24.262	−31.084	−49.263
Inhibition of root elongation									
F	−23.390 ^a^	−35.112 ^d^	−25.607 ^b^	−26.557 ^b^	−43.438 ^c^	−19.846 ^a^	−20.378 ^a^	−24.188 ^b^	−41.184 ^d^
P	−33.634 ^c^	−47.244 ^e^	−54.902 ^f^	−49.520 ^e^	−44.427 ^d^	−50.917 ^f^	−33.615 ^c^	−46.562 ^e^	−53.784 ^f^
Average	−28.512 ^A^	−41.178 ^C^	−40.255 ^B^	−38.039 ^B^	−43.932 ^C^	−35.381 ^A^	−26.997 ^A^	−35.375 ^B^	−47.484 ^C^

C—control soil; F—field dose; P—polluting dose. Homogeneous groups denoted by letters (^a–f^) were calculated separately for each plant, seed germination, and root elongation (two-way ANOVA analysis performed by Tukey at *p* ≤ 0.01). Capital letters (^A–C^) denote homogeneous groups for the test dates separately for each plant.

**Table 8:** Physicochemical properties of soil.

Soil	pH	HAC	EBC	CEC	BS %	C_org_	N_tot_	C:N	Total Exchangeable Cations			
									K^+^	Na^+^	Ca^2+^	Mg^2+^
		mmol^+^ kg^−1^				g kg^−1^			mg kg^−1^			
Sandy loam	7.0	6.4	165.9	172.3	96.28	14.30	0.98	14.6:1	186.0	20.0	2571.40	59.50

pH—soil reaction; HAC—hydrolytic acidity; EBC—sum of exchangeable bases; CEC—sorption capacity; BS—base saturation; C_org_—organic carbon content; N_tot_—total nitrogen content; C:N—ratio of organic carbon content to total nitrogen content.

## Data Availability

Data are available by contacting the authors.

## References

[1] Ali A.A., Desoky E.M., Rady M.M. (2019). Application of azoxystrobin fungicide improves drought tolerance in tomato, via enhancing physio-biochemical and anatomical feature. Int. Lett. Nat. Sci..

[2] Heleno F.F., Rodrigues A.A., Queiroz M.E., Neves A.A., Oliveira A.F., Libardi V.M. (2019). Determination of fungicides in bell pepper using solid-liquid extraction with low temperature partitioning. Microchem. J..

[3] Han L., Liu Y., Fang K., Zhang X., Liu T., Wang F., Wang X. (2020). Azoxystrobin dissipation and its effect on soil microbial community structure and function in the presence of chlorothalonil, chlortetracycline and ciprofloxacin. Environ. Pollut..

[4] Regueiro J., Olguín N., Simal-Gándara J., Suñol C. (2015). Toxicity evaluation of new agricultural fungicides in primary cultured cortical neurons. Environ. Res..

[5] Bollinger E., Zubrod J.P., Konschak M., Sulzer L., Schnurr J., Schreiner V.C., Schulz R., Bundschuh M. (2022). As above, so below? Effects of fungicides on microbial organic matter decomposition are stronger in the hyporheic than in the benthic zone. Limnol. Oceanogr..

[6] Lu T., Zhang Q., Lavoie M., Zhu Y., Ye Y., Yang J., Paerl H.W., Qian H., Zhu Y.G. (2019). The fungicide azoxystrobin promotes freshwater cyanobacterial dominance through altering competition. Microbiome.

[7] Lu C., Hou K., Zhou T., Wang X., Zhang J., Cheng C., Du Z., Li B., Wang J., Wang J. (2023). Characterization of the responses of soil micro-organisms to azoxystrobin and the residue dynamics of azoxystrobin in wheat–corn rotation fields over two years. Chemosphere.

[8] Feng Y., Huang Y., Zhan H., Bhatt P., Chen S. (2020). An overview of strobilurin fungicide degradation: Current status and future perspective. Front. Microbiol..

[9] Edwards P.G., Murphy T.M., Lydy M.J. (2016). Fate and transport of agriculturally applied fungicidal compounds, azoxystrobin and propiconazole. Chemosphere.

[10] Sun M., Wang H., Shi C., Li J., Cai L., Xiang L., Liu T., Goodwin P.H., Chen X., Wang L. (2023). Effect of azoxystrobin on tobacco leaf microbial composition and diversity. Front. Plant Sci..

[11] Yao J., Cui B., Zhao X., Wang Y., Zeng Z., Sun C., Yang D., Liu G., Gao J., Cui H. (2018). Preparation, characterization, and evaluation of azoxystrobin nanosuspension produced by wet media milling. Appl. Nanosci..

[12] Li D., Liu M., Yang Y., Shi H., Zhou J., He D. (2016). Strong lethality and teratogenicity of strobilurins on Xenopus tropicalis embryos: Basing on ten agricultural fungicides. Environ. Pollut..

[13] Pearson B.L., Simon J.M., McCoy E.S., Salazar G., Fragola G., Zylka M.J. (2016). Identification of chemicals that mimic transcriptional changes associated with autism, brain aging and neurodegeneration. Nat. Commun..

[14] Zubrod J.P., Bundschuh M., Arts G., Brühl C.A., Imfeld G., Knäbel A., Payraudeau J.J., Rasmussen J.R., Rohr J., Scharmüller A. (2019). Fungicides: An overlooked pesticide class?. Environ. Sci. Technol..

[15] Yamaguchi T., Mahmood A., Ito T., Kataoka R. (2021). Non-target impact of dinotefuran and azoxystrobin on soil bacterial community and nitrification. Bull. Environ. Contam. Toxicol..

[16] White B.E., Hovenden M.J., Barmuta L.A. (2023). Multifunctional redundancy: Impossible or undetected?. Ecol. Evol..

[17] Zhou Z., Wang C., Luo Y. (2020). Meta-analysis of the impacts of global change factors on soil microbial diversity 827 and functionality. Nat. Commun..

[18] Mpofu E., Alias A., Tomita K., Suzuki-Minakuchi C., Tomita K., Chakraborty J., Malon M., Ogura Y., Takikawa H., Okada K. (2021). Azoxystrobin amine: A novel azoxystrobin degradation product from *Bacillus licheniformis* strain TAB7. Chemosphere.

[19] Delgado-Baquerizo M., Oliverio A.M., Brewer T.E., Benavent-González A., Eldridge D.J., Bardgett R.D., Maestre F.T., Singh B.K., Fierer N. (2018). A global atlas of the dominant bacteria found in soil. Science.

[20] Zhang Q., Zhu D., Ding J., Zheng F., Zhou S., Lu T., Zhu Y.-G., Qian H. (2019). The fungicide azoxystrobin perturbs the gut microbiota community and enriches antibiotic resistance genes in *Enchytraeus crypticus*. Environ. Int..

[21] Zhu L., Chen Y., Sun R., Zhang J., Hale L., Dumack K., Geisen S., Deng Y., Zhu B., Li Y. (2023). Resource-dependent biodiversity and potential 833 multi-trophic interactions determine belowground functional trait stability. Microbiome.

[22] Silva V., Mol H.G., Zomer P., Tienstra M., Ritsema C.J., Geissen V. (2019). Pesticide residues in European agricultural soils–A hidden reality unfolded. Sci. Total Environ..

[23] Meena R.S., Kumar S., Datta R., Lal R., Vijayakumar V., Brtnicky M., Sharma M.P., Yadav G.S., Jhariya M.K., Jangir C.K. (2020). Impact of agrochemicals on soil microbiota and management: A review. Land.

[24] Cycoń M., Mrozik A., Piotrowska-Seget Z. (2017). Bioaugmentation as a strategy for the remediation of pesticide-polluted soil: A review. Chemosphere.

[25] Sharma A., Shukla A., Attri K., Kumar M., Kumar P., Suttee A., Singh G., Barnwal R.P., Singla N. (2020). Global trends in pesticides: A looming threat and viable alternatives. Ecotoxicol. Environ. Saf..

[26] Singh S., Rawat M., Malyan S.K., Singh R., Tyagi V.K., Singh K., Kashyap S., Kumar S., Sharma M., Panday B.K. (2023). Global distribution of pesticides in freshwater resources and their remediation approaches. Environ. Res..

[27] Verdenelli R.A., Dominchin M.F., Barbero F.M., Pérez-Brandán C., Aoki A., Gil S.V., Meriles J.M. (2023). Effect of two broad-spectrum fungicides on the microbial communities of a soil subjected to different degrees of water erosion. Appl. Soil Ecol..

[28] Da Rocha A.G., Pitombo L.M., Bresolin J.D., da Silva W.T.L., Espindola E.L.G., de Menezes Oliveira V.B. (2020). Single and com-bined toxicity of the pesticides abamectin and difenoconazole on soil microbial activity and *Enchytraeus crypticus* population. SN Appl. Sci..

[29] Chamberlain L.A., Aguayo T., Zerega N.J.C., Dybzinski R., Egerton-Warburton L.M. (2022). Rapid improvement in soil health following the conversion of abandoned farm fields to annual or perennial agroecosystems. Front. Sustain. Food Syst..

[30] Roman D.L., Voiculescu D.I., Filip M., Ostafe V., Isvoran A. (2021). Effects of triazole fungicides on soil microbiota and on the activities of enzymes found in soil: A review. Agriculture.

[31] Filimon M.N., Voia S.O., Vlădoiu D.L., Isvoran A., Ostafe V. (2015). Temperature dependent effect of difenoconazole on enzymatic activity from the soil. J. Serbian Chem. Soc..

[32] Zhang D., Wu Y., Zhang X., Zhu Y. (2017). Persistence of myclobutanil and its impact on soil microbial biomass C and dehydrogenase enzyme activity in tea orchard soils. Eurasian J. Soil Sci..

[33] Satapute P., Kamble M.V., Adhikari S.S., Jogaiah S. (2019). Influence of triazole pesticides on tillage soil microbial populations and metabolic changes. Sci. Total Environ..

[34] Pathak V.M., Verma V.K., Rawat B.S., Kaur B., Babu N., Sharma A., Dewali S., Yadav M., Kumari R., Singh S. (2022). Current status of pesticide effects on environment, human health and it’s eco-friendly management as bioremediation: A comprehensive review. Front. Microbiol..

[35] Yengkokpam P., Mazumder P.B. (2020). Phytotoxicity of malathion (PM) and tatafen (PTF) towards *Solanum melongena* L. cv. Longai: A case study. Plant Phys. Rep..

[36] Bhatt P., Huang Y., Zhang W., Sharma A., Chen S. (2020). Enhanced cypermethrin degradation kinetics and metabolic pathway in *Bacillus thuringiensis* strain SG4. Microorganisms.

[37] Bhatt P., Huang Y., Rene E.R., Kumar A.J., Chen S. (2020). Mechanism of allethrin biodegradation by a newly isolated *Sphingomonas trueperi* strain CW3 from wastewater sludge. Bioresour. Technol..

[38] Conde-Avila V., Ortega-Martínez L.D., Loera O., El Kassis E.G., Dávila J.G., Valenzuela C.M., Armendáriz B.P. (2021). Pesticides degradation by immobilised microorganisms. Int. J. Environ. Anal. Chem..

[39] Alexandrino D.A., Mucha A.P., Almeida C.M.R., Carvalho M.F. (2020). Microbial degradation of two highly persistent fluorinated fungicides-epoxiconazole and fludioxonil. J. Hazard. Mater..

[40] Dennis P.G., Kukulies T., Forstner C., Orton T.G., Pattison A.B. (2018). The effects of glyphosate, glufosinate, 620 paraquat and paraquat-diquat on soil microbial activity and bacterial, archaeal and nematode 621 diversity. Sci. Rep..

[41] Storck V., Nikolaki S., Perruchon C., Chabanis C., Sacchi A., Pertile G., Baguelin C., Karas P.A., Spor A., Devers-Lamrani M. (2018). Lab to field assessment of the ecotoxicological impact of chlorpyrifos, isoproturon, or tebuconazole on the diversity and composition of the soil bacterial community. Front. Microbiol..

[42] Wang F., Li X., Zhu L., Du Z., Zhang C., Wang J., Wang J., Lv D. (2018). Responses of soil microorganisms and enzymatic activities to azoxystrobin in Cambisol. Pol. J. Environ. Stud..

[43] Huang X., He J., Yan X., Hong Q., Chen K., He Q., Zhang L., Liu X., Chuang S., Li S. (2017). Microbial catabolism of chemical herbicides: 647 Microbial resources, metabolic pathways and catabolic genes. Pestic. Biochem. Physiol..

[44] Thiour-Mauprivez C., Martin-Laurent F., Calvayrac C., Barthelmebs L. (2019). Effects of herbicide on non-target microorganisms: Towards a new class of biomarkers?. Sci. Total Environ..

[45] Święciło A., Krzepiłko A., Michałek S. (2018). Evaluation of azoxystrobin toxicity to saprophytic fungi and radish in the early stages of growth. Ecol. Chem. Eng. A.

[46] Andreolli M., Lampis S., Tosi L., Marano V., Zapparoli G. (2023). Fungicide sensitivity of grapevine bacteria with plant growth-promoting traits and antagonistic activity as non-target microorganisms. World J. Microbiol. Biotechnol..

[47] Baćmaga M., Wyszkowska J., Borowik A., Kucharski J. (2022). Effects of tebuconazole application on soil microbiota and enzymes. Molecules.

[48] Sułowicz S., Cycoń M., Piotrowska-Seget Z. (2016). Non-target impact of fungicide tetraconazole on microbial communities in soils with different agricultural management. Ecotoxicology.

[49] Podbielska M., Kus-Liśkiewicz M., Jagusztyn B., Szpyrka E. (2023). Effect of microorganisms on degradation of fluopyram and tebuconazole in laboratory and field studies. Environ. Sci. Pollut. Res..

[50] Cycoń M., Markowicz A., Piotrowska-Seget Z. (2013). Structural and functional diversity of bacterial community in soil treated with the herbicide napropamide estimated by the DGGE, CLPP and r/K-strategy approaches. Appl. Soil Ecol..

[51] Baćmaga M., Wyszkowska J., Kucharski J. (2019). Biostimulation as a process aiding tebuconazole degradation in soil. J. Soils Sediments.

[52] Lipińska A., Wyszkowska J., Kucharski J. (2021). Microbiological and biochemical activity in soil contaminated with pyrene subjected to bioaugmentation. Water Air Soil Pollut..

[53] Chen X., He S., Liang Z., Li Q.X., Yan H., Hu J., Liu X. (2018). Biodegradation of pyraclostrobin by two microbial communities from Hawaiian soils and metabolic mechanism. J. Hazard. Mater..

[54] Luong T.T., Nguyen T.H.T., Nguyen T.D., Pham T.H.T., Ho T.T., Nguyen N.L. (2024). Degradation of triazole fungicides by plant growth-promoting bacteria from contaminated agricultural soil. J. Microbiol. Biotechnol..

[55] Clinton B., Warden A.C., Haboury S., Easton C.J., Kotsonis S., Taylor M.C., Oakeshott J.G., Russell R.J., Scott C. (2011). Bacterial degradation of strobilurin fungicides: A role for a promiscuous methyl esterase activity of the subtilisin proteases?. Biocatal. Biotransform..

[56] Howell C.C., Semple K.T., Bending G.D. (2014). Isolation and characterisation of azoxystrobin degrading bacteria from soil. Chemosphere.

[57] Hocinat A., Boudemagh A. (2016). Biodegradation of commercial ortiva fungicide by isolated actinomycetes from the activated sludge, Desalin. Water Treat..

[58] Feng Y., Zhang W., Pang S., Lin Z., Zhang Y., Huang Y., Bhatt P., Chen S. (2020). Kinetics and new mechanism of azoxystrobin biodegradation by an *Ochrobactrum anthropi* strain SH14. Microorganisms.

[59] Kenarova A., Boteva S. (2023). Fungicides in agriculture and their side effects on soil enzyme activities: A review. Bulg. J. Agric. Sci..

[60] Wang X., Lu Z., Miller H., Liu J., Hou Z., Liang S., Zhao H., Borch T. (2020). Fungicide azoxystrobin induced changes on the soil microbiome. Appl. Soil Ecol..

[61] Boteva S.B., Kenarova A.E., Petkova M.R., Georgieva S.S., Chanev C.D., Radeva G.S. (2022). Soil enzyme activities after application of fungicide QuadrisR at increasing concentration rates. Plant Soil Environ..

[62] Roman D.L., Matica M.A., Ciorsac A., Boros B.V., Isvoran A. (2023). The effects of the fungicide myclobutanil on soil enzyme activity. Agriculture.

[63] Amaro A.C.E., Ramos A.R.P., Macedo A.C., Ono E.O., Rodrigues J.D. (2018). Effects of the fungicides azoxystrobin, pyraclostrobin and boscalid on the physiology of Japanese cucumber. Sci. Hortic..

[64] Chiu-Yueh L.A.N., Kuan-Hung L.I.N., Huang W.D., Chang-Chang C.H.E.N. (2019). Physiological effects of the fungicide azoxystrobin on wheat seedlings under extreme heat. Not. Bot. Horti Agrobot. Cluj-Napoca.

[65] Emam S.S., Abd-Eldaim F.A. (2022). Effect of difenoconazole and azoxystrobin on wheat and radish seeds germination and tomato seedling growth. Egypt. Acad. J. Biol. Sci..

[66] Macar O., Kalefetoğlu Macar T., Yalçın E., Çavuşoğlu K. (2022). Acute multiple toxic effects of trifloxystrobin fungicide on *Allium cepa* L.. Sci. Rep..

[67] IUSS Working Group World Reference Base for Soil Resources (2014). International Soil Classification System for Naming Soils and Creating Legends for Soil Maps.

[68] Borowik A., Wyszkowska J., Wyszkowski M. (2017). Resistance of aerobic microorganisms and soil enzyme response to soil contamination with Ekodiesel Ultra fuel. Environ. Sci. Pollut. Res..

[69] Ministry of Agriculture and Rural Development The Register of Authorized Plant Protection Products, 2024—Update 21 March 2024. https://www.gov.pl.

[70] (2004). ISIS-Draw, MDL, Version 2.3. https://mdl-isis-draw.software.informer.com/2.3/.

[71] Lewis K., Tzilivakis J.G. (2017). Development of a data set of pesticide dissipation rates in/on various plant matrices for the Pesticide Properties Database (PPDB). Data.

[72] Kucharski J., Tomkiel M., Baćmaga M., Borowik A., Wyszkowska J. (2016). Enzyme activity and microorganisms diversity in soil contaminated with the Boreal 58 WG herbicide. J. Environ. Sci. Health B.

[73] Wyszkowska J., Boros-Lajszner E., Kucharski J. (2024). The impact of soil contamination with lead on the biomass of maize intended for energy purposes, and the biochemical and physicochemical properties of the soil. Energies.

[74] Baćmaga M., Wyszkowska J., Kucharski J. (2016). The effect of the Falcon 460 EC fungicide on soil microbial communities, enzyme activities and plant growth. Ecotoxicology.

[75] Lipińska A., Wyszkowska J., Kucharski J. (2015). Diversity of organotrophic bacteria, activity of dehydrogenases and urease as well as seed germination and root growth *Lepidium sativum*, *Sorghum saccharatum* and *Sinapis alba* under the influence of polycyclic aromatic hydrocarbons. Environ. Sci. Pollut. Res..

[76] Starchel R., Wyszkowska J., Baćmaga M. (2020). Bioaugmentation of soil contaminated with zinc. Water Air Soil Pollut..

[77] Tamura K., Stecher G., Kumar S. (2021). MEGA11: Molecular evolutionary genetics analysis version 11. Mol. Biol. Evol..

[78] Wyszkowska J., Borowik A., Zaborowska M., Kucharski J. (2023). The usability of sorbents in restoring enzymatic activity in soils polluted with petroleum-derived products. Materials.

[79] Borowik A., Wyszkowska J., Kucharski J. (2022). Bacteria and soil enzymes supporting the valorization of forested soils. Materials.

[80] Wyszkowska J., Boros-Lajszner E., Kucharski J. (2022). Calorific value of *Festuca rubra* biomass in the phytostabilization of soil contaminated with nickel, cobalt and cadmium which disrupt the microbiological and biochemical properties of soil. Energies.

[81] Lipińska A., Kucharski J., Wyszkowska J. (2019). Activity of phosphatases in soil contaminated with PAHs. Water Air Soil Pollut..

[82] TIBCO Software Inc. (2017). Statistica. Data Analysis Software System, Version 13. https://www.statistica.com.

